# HER2-positive gastric cancer

**DOI:** 10.1007/s10120-013-0252-z

**Published:** 2013-04-07

**Authors:** Narikazu Boku

**Affiliations:** Department of Clinical Oncology, St. Marianna University School of Medicine, 2-16-1 Sugao Miyamae-ku, Kawasaki, Kanagawa Japan

**Keywords:** Stomach neoplasms, Trastuzumab, Receptor erbB-2, HER-2 proto-oncogene protein

## Abstract

Human epidermal growth factor receptor 2 (HER2) is involved in the pathogenesis and poor outcomes of several types of cancer, including advanced gastric and gastroesophageal junction cancer. Molecular-targeted drugs, such as trastuzumab, which prolong overall survival and progression-free survival in HER2-positive breast cancer, may also be beneficial in patients with HER2-positive gastric cancer. Several studies have examined this possibility, such as the Trastuzumab for Gastric Cancer trial. In this context, the first part of this review provides an update on our knowledge of HER2 in breast and gastric cancer, including the detection and prognostic relevance of HER2 in gastric cancer. The second part of the review discusses the results of pivotal clinical trials that examined the potential for using trastuzumab to treat this disease. This section also summarizes the trials that have been conducted or that are underway to determine the optimal uses of trastuzumab in gastric cancer, including its use as monotherapy and continuation beyond disease progression. The final section discusses the future prospects of other anti-HER2 drugs, including lapatinib, trastuzumab emtansine, and pertuzumab, for the treatment of HER2-positive gastric cancer. The introduction of trastuzumab led to the establishment of a new disease entity, “HER2-positive gastric cancer,” similar to HER2-positive breast cancer. It is expected that more anti-HER2 drugs will be developed and introduced into clinical practice to treat HER2-positive cancers, including gastric cancer.

## Introduction

Up to 30 % of breast cancers overexpress human epidermal growth factor receptor 2 (HER2, c-erbB2), and HER2 positivity is associated with significantly worse outcomes than HER2-negative breast cancer [1]. Trastuzumab, a monoclonal antibody directed against HER2, was one of the first molecular-targeted drugs to be developed and was originally introduced for the treatment of HER2-positive metastatic breast cancer. Its approval in this setting was based on two pivotal studies, which showed the efficacy of trastuzumab administered with paclitaxel [2] or trastuzumab alone as first-line therapy [3]. Studies have since demonstrated its efficacy for treating early breast cancer when used with either adjuvant [4–6] or neoadjuvant [7–9] chemotherapy, conferring prolonged survival and improved outcomes compared with the established therapies using cytotoxic agents alone. Over the last decade, trastuzumab has revolutionized the treatment of HER2-positive breast cancer and improved its outcomes [10]. Based on these findings, trastuzumab is now considered a key drug for treating HER2-positive breast cancer, which has been established as a major disease subtype of breast cancer.

With increasing understanding of the molecular biology of HER2, and the availability of genomics and proteomics analyses, it has now been recognized that HER2 is implicated in other severe forms of cancer, notably gastric cancer. Therefore, the aims of this review are to provide an update on our knowledge of HER2 in the context of gastric cancer and to describe the clinical trials that have examined the potential of using trastuzumab to treat this disease, such as the Trastuzumab for Gastric Cancer (ToGA) trial [11], or are currently underway.

## Gastric cancer and the biological relevance of HER2

HER2 is a proto-oncogene encoded by *ERBB2* on chromosome 17. It is a member of the HER family and consists of four plasma membrane-bound receptor tyrosine kinases that transmit extracellular signals to initiate cellular signaling pathways via mitogen-activated protein kinase, phosphoinositide 3-kinase, phospholipase C, protein kinase C, and signal transducer and activator of transcription. Following early studies [12–14], it has now become clear that HER2 is expressed in many tissues, including the breast, gastrointestinal tract, kidney, and heart. Its major role in these tissues is to promote cell proliferation and suppress apoptosis, which may facilitate excessive/uncontrolled cell growth and tumorigenesis [15–17].

Overexpression/amplification of HER2/*ERBB2* in breast cancer, resulting in HER2-positive subtypes, is associated with very poor prognosis compared with HER2-negative breast cancer [1, 18]. HER2-positive breast cancer is also associated with increased risk of local growth and distant metastasis. Many studies, including several conducted in Japan, have demonstrated that HER2 is also present in other cancers, particularly in gastric cancer [19–22]. Consequently, many studies have evaluated the relationship between HER2 status and prognosis in patients with gastric cancer [23–33]. Unlike in breast cancer, the studies in gastric cancer to date have yielded inconsistent findings regarding the prognostic relevance of HER2. Some showed that HER2 positivity was associated with a significantly worse prognosis [23, 26, 28, 31, 32], whereas others found no association between HER2 status and prognosis [25, 33], or that median overall survival was longer in HER2-positive than in HER2-negative patients [24, 25]. Therefore, the relationship between HER2 status and prognosis of gastric cancer patients remains controversial.

In the context of breast cancer, the American Society of Clinical Oncology/College of American Pathologists noted that as many as 20 % of HER2 tests performed may be inaccurate [34], which may also influence studies attempting to determine the frequency of HER2-positive gastric cancer. Because of differences in the examination method and objective criteria, the frequency of HER2-positive gastric cancer varies considerably between studies, ranging from 6.0 to 29.5 % in earlier studies (Table 1). In an effort to address these inconsistencies, the investigators in the ToGA trial conducted a validation study to assess the immunohistochemistry (IHC) and fluorescence in situ hybridization (FISH) protocols for testing HER2 status in advanced gastric cancer [35]. Tissue specimens from 3,807 patients in 24 countries were collected and analyzed at a central laboratory using both IHC and FISH methods [11, 36]. HER2 status was defined as positive (IHC 3+ or FISH-positive) based on the surgical or biopsy specimen staining patterns (Table 2). Notably, there were no marked racial differences in HER2 expression; instead, differences in HER2 expression were mainly attributed to the site of the primary tumor (gastric vs. gastroesophageal junction) and histological type [36]. The criteria for HER2 status also differ between breast cancer and gastric cancer because of differences in the IHC staining pattern for HER2 between these sites [35].

**Table 1:** Prevalence of HER2 positivity in patients with gastric cancer

Study	Country	*n*	Determination of HER2 status	HER2-positive (%)	Prognosis
Takehana et al. [76]	Japan	352	IHC 2+/IHC 3+	8.2	n/a
Tanner et al. [32]	Finland	231	CISH+	36.6	++
Park et al. [31]	Korea	182	IHC 2+/IHC 3+	15.9	++
			CISH +/FISH+	3.8	
Yano et al. [77]	Japan	200^a^	IHC 2 +/IHC 3+	23.0	n/a
		199^a^	FISH	27.1	
Kim et al. [78]	Korea	248	EMA label^b^	6.0	–
Matsubara et al. [79]	Japan	87	>10 %	18.0	–
Barros-Silva et al. [80]	Portugal	463	IHC 2+/IHC 3+	9.3	+
			EMA label^b^	8.0	
Yan et al. [81]	Singapore	128	FISH+	11.7	+
			IHC 3+	9.4	
Yan et al. [82]	China	145	EMA label^b^	10.3	+
Lee et al. [83]	Australia	178	EMA label^b^	20.2	n/a
Liu et al. [84]	China	775	EMA label^b^	12.1	+
Giuffrè et al. [85]	Italy	109	EMA label^b^	21.1	++
Tsapralis et al. [86]	Greece	120	IHC 2+/IHC 3+	16.6	–
			ISH+	15.8	
Terashima et al. [33]	Japan	829	IHC 3+ or IHC 2 +/DISH+	9.0	–
Wang et al. [87]	China	102	EMA label^b^	14.7	–
Kim et al. [28]	Korea	111	FISH+	9.0	++
Halon et al. [88]	Poland	78	IHC 2+/IHC 3+	29.5	–

*n/a* not applicable, — no association was found between HER2 expression and prognosis, *+* HER2 expression was partially associated with poor prognosis, *++* HER2 expression was associated with poor prognosis, *EMA* European Medicines Agency

^a^Invasive intestinal cancer only

^b^IHC 3+ or IHC 2+/FISH-positive

**Table 2:** Immunohistochemistry scoring for HER2 expression in gastric and gastroesophageal junction cancer used in the ToGA trial [11]

Score	Surgical specimen staining pattern	Biopsy specimen staining pattern	HER2 overexpression assessment
0	No reactivity or membranous reactivity in <10 % of tumor cells	No reactivity or no membranous reactivity in any tumor cell	Negative
1+	Faint or barely perceptible membranous reactivity in ≥10 % of tumor cells; cells are reactive only in part of their membrane	Tumor cell cluster with a faint or barely perceptible membranous reactivity irrespective of percentage of tumor cells stained	Negative
2+	Weak to moderate complete, basolateral or lateral membranous reactivity in ≥10 % of tumor cells	Tumor cell cluster with a weak to moderate complete, basolateral or lateral membranous reactivity irrespective of percentage of tumor cells stained	Equivocal
3+	Strong complete, basolateral or lateral membranous reactivity in ≥10 % of tumor cells	Tumor cell cluster with a strong complete, basolateral or lateral membranous reactivity irrespective of percentage of tumor cells stained	Positive

Reprinted with permission from Elsevier Ltd

HER2 status is mainly assessed by IHC or FISH using biopsy or surgical specimens. Based on the results of the ToGA study, trastuzumab was approved for HER2-positive gastric cancer, which is defined as IHC 3+ or FISH-positive in the USA and Japan. Conversely, HER2-positive gastric cancer is defined as IHC 3+ or as IHC 2 + plus FISH-positive in Europe [37]. The guidelines for HER2 testing of gastric cancer developed by the Japanese Society of Pathology [38] recommend that HER2 testing should be routinely performed in patients with metastatic or recurrent gastric cancer. The testing algorithms developed for HER2 involve IHC first, followed by FISH for IHC 2+ patients. In order to confirm the frequency of HER2-positive gastric cancer found in ToGA, a prospective study is now underway to determine the prevalence of HER2-positive cancer in Japanese patients [39].

The results of the studies described above have provided a clear rationale for the use of drugs targeting HER2, such as trastuzumab, to treat gastric cancer. Accordingly, the aims of the next part of this review are to summarize the results of studies in this setting and identify opportunities for further research.

## Efficacy and safety of trastuzumab in gastric cancer: the ToGA trial

The ToGA trial was a prospective, phase 3, open-label trial in which patients with HER2-positive advanced gastric or gastroesophageal junction cancer were randomly allocated to receive either trastuzumab in combination with chemotherapy or chemotherapy alone [11]. Chemotherapy was given every 3 weeks for six cycles. Trastuzumab was administered at a dose of 8 mg/kg on day 1 of the first cycle and then at 6 mg/kg every 3 weeks until disease progression, unacceptable toxicity, or withdrawal of consent. Overall, 3,803 patients were screened for the study, 810 were HER2-positive (based on the criteria listed in Table 2), 594 were randomized, and 584 received study treatment and were analyzed. The general characteristics of patients in the trastuzumab plus chemotherapy (*n* = 294) and chemotherapy-alone (*n* = 290) groups were similar, including age (59.4 vs. 58.5 years), sex (proportion of men: 77 vs. 75 %), chemotherapy regimen (capecitabine and cisplatin: 87 vs. 88 %; fluorouracil and cisplatin: 13 vs. 12 %), and primary tumor site (stomach: 90 vs. 83 %; gastroesophageal junction: 20 vs. 17 %). Overall, 97 % of patients in both groups had metastatic disease at study entry, and just under half were classified as FISH-positive/IHC 3 + (45 vs. 43 %).

**Fig. 1:**
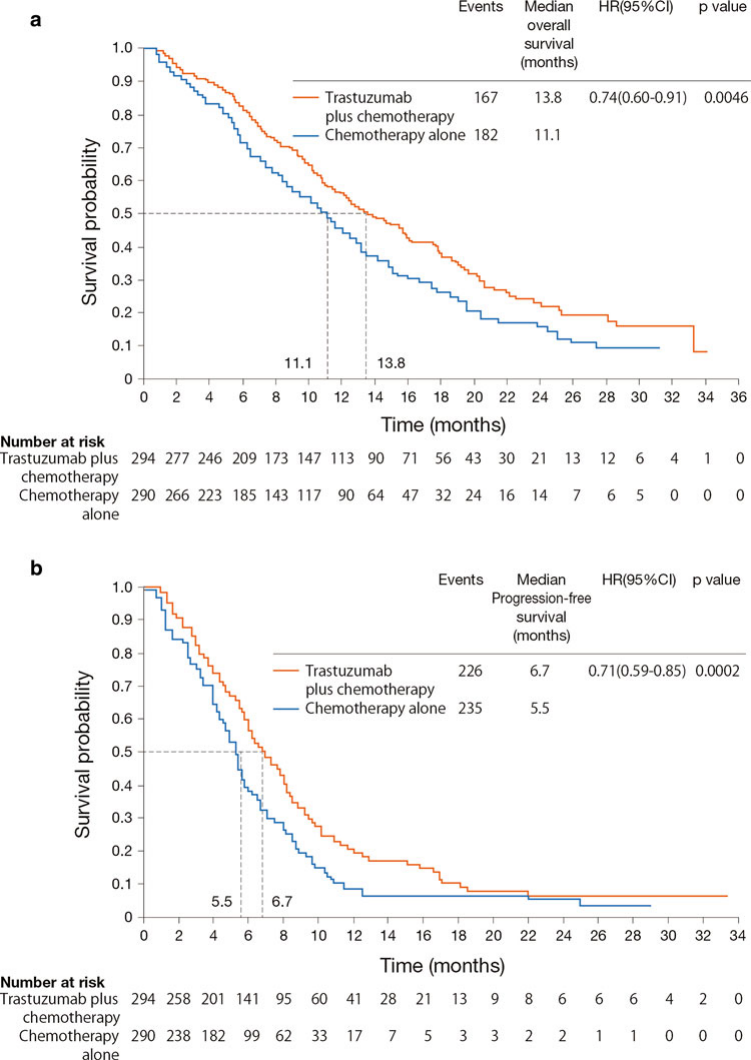
Median survival (**a**) and progression-free survival (**b**) in the ToGA trial [11]. *HR* hazard ratio, *CI* confidence interval. Reprinted with permission from Elsevier Ltd

The primary endpoint of the study was overall survival, which was defined as the time from randomization to death from any cause. As shown in Fig. 1a, overall survival was significantly longer in patients receiving trastuzumab plus chemotherapy compared with chemotherapy alone, with an increase of 2.7 months in median overall survival [13.8 vs. 11.1 months; hazard ratio (HR): 0.74; 95 % confidence interval (CI): 0.60–0.91; *P* = 0.0046]. Progression-free survival was also extended by trastuzumab plus chemotherapy compared with chemotherapy alone (6.7 vs. 5.5 months; HR: 0.71; 95 % CI: 0.59–0.85; *P* = 0.0002) (Fig. 1b). The overall response rate in the trastuzumab plus chemotherapy group was 47 % (complete response: 5 %; partial response: 42 %) and was significantly greater than that in the chemotherapy-alone group (35 %; *P* = 0.0017; complete response: 2 %; *P* = 0.0599; partial response: 32 %; *P* = 0.0145). The duration of response (6.9 vs. 4.8 months; *P* < 0.0001) was also significantly longer in the trastuzumab plus chemotherapy group.

**Fig. 2:**
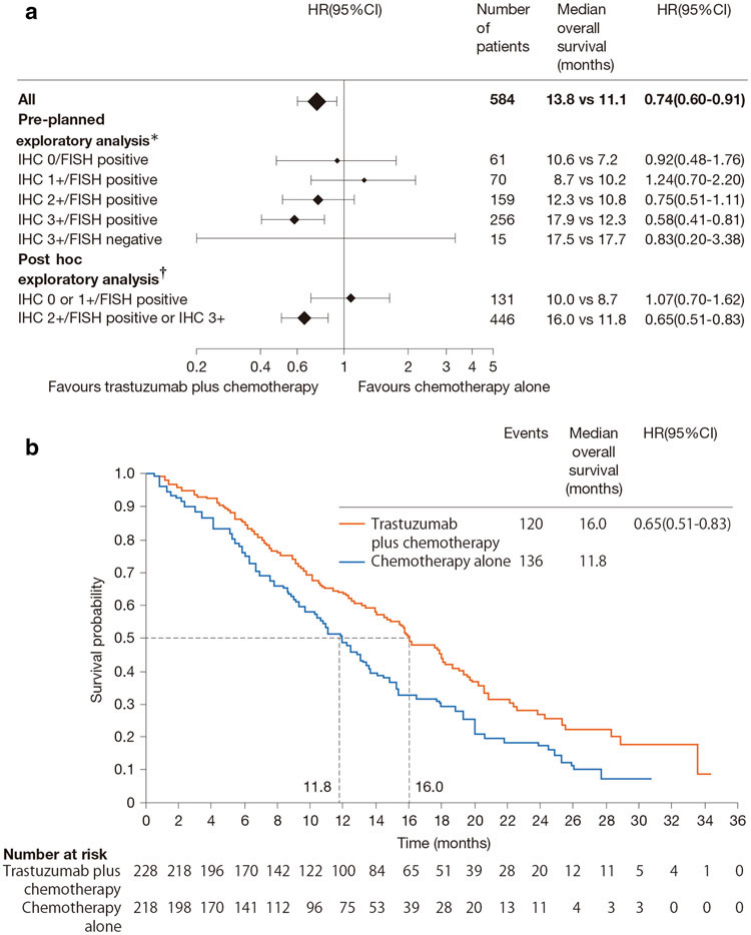
Results of the ToGA trial [11]. **a** Pre-planned exploratory and post hoc exploratory analyses of patients stratified by HER2 status. *n = 561; patients with no immunohistochemistry (IHC) data (*n* = 7) or IHC 3+ tumors with no fluorescence in situ hybridization (FISH) data (*n* = 16) were excluded from the analysis. ^†^ *n* = 577; patients with no IHC data were excluded from the analysis. **b** Overall survival in patients with higher HER2 expression (IHC 2+ and FISH-positive tumors or IHC 3+ tumors). *HR* hazard ratio, *CI* confidence interval. Reprinted with permission from Elsevier Ltd

Pre-planned and post hoc exploratory analyses of subgroups of patients also revealed that overall survival was longer in patients with higher HER2 expression, as determined by IHC and FISH (i.e., IHC 3+ or IHC 2+/FISH-positive), than in patients with lower HER2 expression (i.e., IHC 0 or 1+/FISH-positive). Among patients with higher HER2 expression, survival was significantly extended by trastuzumab in combination with chemotherapy compared with chemotherapy alone (16.0 vs. 11.8 months; HR: 0.65; 95 % CI: 0.51–0.83) (Fig. 2). Based on the results of these tests, trastuzumab therapy is strongly recommended for patients with IHC 3+ or IHC 2+/FISH-positive (high HER2 expression) in clinical practice in Japan.

The incidence of adverse events was similar in both groups, with grade 3 or 4 events occurring in 68 % of patients in both groups, the most common of which were neutropenia, anemia, nausea, and vomiting.

The results of this study showed that trastuzumab in combination with chemotherapy significantly improved overall survival in patients with HER2-positive advanced gastric or gastroesophageal cancer compared with chemotherapy alone, and this improvement was particularly significant in patients with high HER2 expression. It is also notable that trastuzumab did not increase the incidence of adverse events associated with chemotherapy and that the rate of cardiac events (e.g., cardiac failure and decreases in left ventricular ejection fraction) was low.

## Trastuzumab as maintenance therapy

In terms of the optimal treatment of patients with HER2-positive cancer, several regimens using trastuzumab have been proposed and tested in clinical settings. Trastuzumab monotherapy is used as maintenance therapy for patients with breast cancer based on the study by Vogel et al. [3], who reported an objective response rate of 26 %, which was driven by the response rate in patients with HER2 expression scored as IHC 3+ (35 %); no patient with IHC 2+ expression showed a response. In that study, trastuzumab was administered with a 4 mg/kg loading dose followed by 2 mg/kg dose weekly or a 8 mg/kg loading dose followed by 4 mg/kg weekly. In the Herceptin Adjuvant (HERA) trial, women with HER2-positive advanced breast cancer were randomly assigned to 1 or 2 years of treatment with trastuzumab or observation after locoregional therapy and at least four cycles of neoadjuvant or adjuvant chemotherapy [4]. The primary article presented the results for the 1-year and observation groups, where Kaplan–Meier analysis showed that 1 year of trastuzumab maintenance therapy was associated with significantly greater disease-free survival (85.8 vs. 77.4 %; *P* < 0.0001) and distant recurrence-free survival (90.6 vs. 82.8 %; *P* < 0.0001), although not overall survival (96.0 vs. 95.1 %; *P* = 0.26), compared with observation alone, with a median follow-up of 1 year [4]. However, at a median follow-up of 2 years, the risk of death was significantly lower in patients treated with trastuzumab for 1 year compared with observation alone (HR: 0.66; 95 % CI: 0.47–0.91; *P* = 0.0115), as was the risk of a disease-free survival event (HR: 0.64; 95 % CI: 0.54–0.76; *P* < 0.001) [40]. At a median follow-up of 4 years, there remained a significant disease-free survival benefit of 1 year of trastuzumab therapy compared with observation alone (HR: 0.76; 95 % CI: 0.66–0.87; *P* < 0.0001), although the risk of death was no longer significantly different (HR: 0.85; 95 % CI: 0.70–1.04; *P* = 0.11) [41]. Disease-free survival and overall survival were also significantly greater at a median follow-up of 8 years in patients given 1 year of trastuzumab maintenance therapy compared with observation alone (HR: 0.76, *P* < 0.0001; and HR: = 0.76, *P* = 0.0005, respectively), demonstrating the durable effects of trastuzumab on survival and preventing disease recurrence [42].

Initial data for patients allocated to 2 years of trastuzumab maintenance therapy were published in 2012 [42], with a median follow-up of 8 years. In that analysis, the unadjusted HR for any disease-free survival event in the 2- vs. 1-year trastuzumab groups was 0.99 (95 % CI: 0.85–1.14; *P* = 0.86). Overall survival was also comparable in both groups (HR: 1.05; 95 % CI: 0.86–1.28; *P* = 0.63). Based on these data from the HERA trial, the authors concluded that 1 year of trastuzumab maintenance therapy should be considered as the standard of care for patients with HER2-positive advanced breast cancer.

To expand these breast cancer findings of trastuzumab monotherapy into the setting of gastric cancer, a pilot study was conducted in which patients who progressed while on chemotherapy for metastatic or locally advanced HER2-positive gastric cancer were treated with trastuzumab monotherapy [43]. However, the study only involved four patients; therefore, additional studies are needed to confirm the potential of trastuzumab monotherapy.

**Fig. 3:**
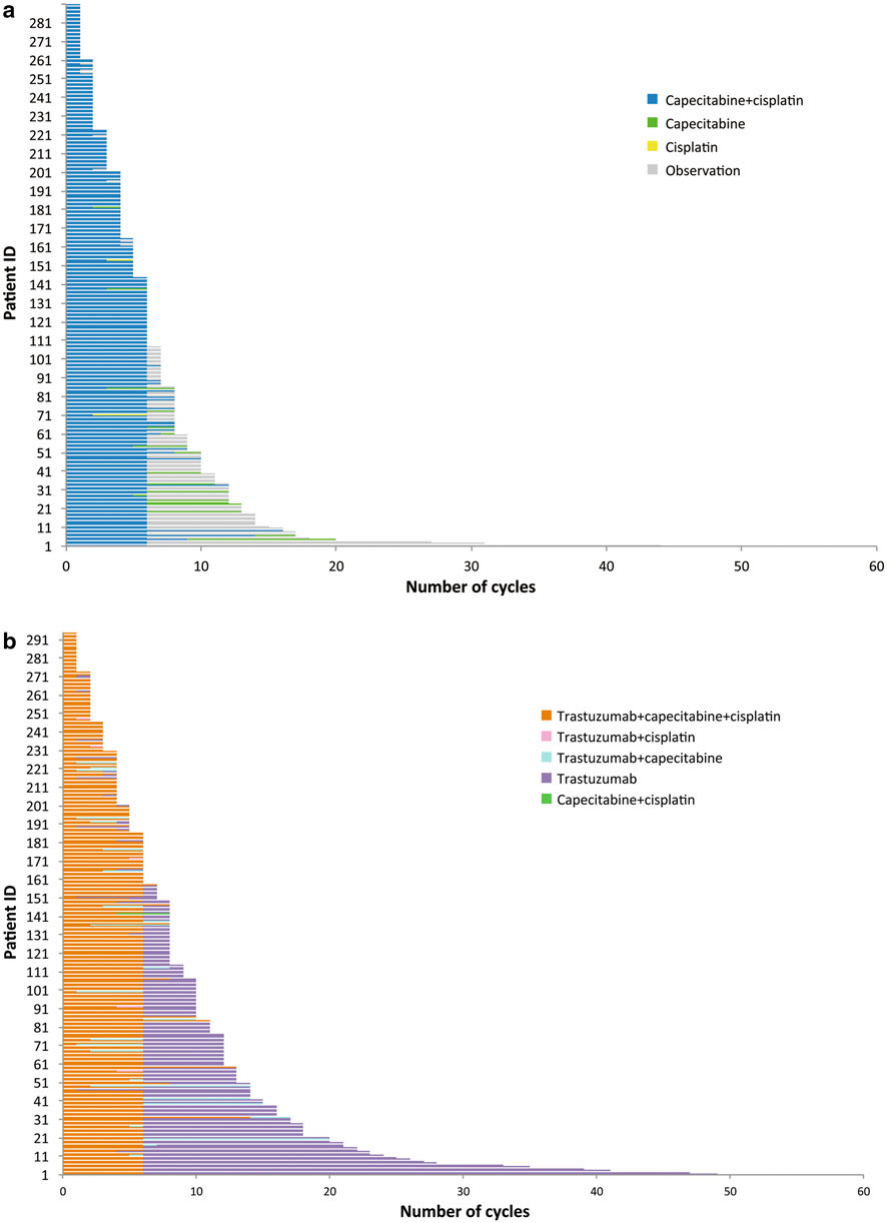
Number of cycles of combination drugs administered in individual patients enrolled in the ToGA trial [11] in the control (**a**) or trastuzumab (**b**) groups

In the early part of the ToGA trial, patients in the trastuzumab group received six cycles of chemotherapy in combination with trastuzumab and then continued trastuzumab monotherapy until disease progression. Patients in the trastuzumab group could also continue trastuzumab monotherapy until disease progression, even if unacceptable toxicity of chemotherapy occurred during the planned six cycles. By contrast, patients in the control group entered an observation period after completion of six cycles of chemotherapy or after withdrawal of chemotherapy during the planned six cycles. Since August 2007, extended cycles of chemotherapy were allowed after considering the risk/benefit ratio for each patient. Thus, the ToGA protocol allowed for trastuzumab to be continued while chemotherapy was discontinued or the doses reduced, even if notable toxicities occurred that were possibly caused by chemotherapy. In order to estimate the efficacy of trastuzumab as maintenance therapy in the ToGA trial, the duration of trastuzumab monotherapy in the trastuzumab arm was compared with the observation period of the chemotherapy-alone arm. As shown in Fig. 3, 159 patients (54.1 %) received trastuzumab monotherapy after combination therapy with trastuzumab (*n* = 294) [44]. Only 97/290 patients (33.4 %) in the chemotherapy-alone group experienced chemotherapy-free periods. Reasons for switching to trastuzumab monotherapy included completion of six cycles of chemotherapy (125/159, 79 %), physician’s judgment (22/159, 14 %), or an adverse event associated with chemotherapy (12/159, 8 %). Although statistical comparison between the trastuzumab monotherapy periods and the chemotherapy-free periods was not performed, the former was apparently longer than the latter. Continuation of trastuzumab after discontinuation of chemotherapy likely contributed to the prolonged survival in the trastuzumab plus chemotherapy group. The ToGA trial therefore indicated that starting trastuzumab in combination with chemotherapy and then continuing trastuzumab monotherapy until disease progression extended overall survival in patients with gastric cancer.

The survival advantage of second-line chemotherapy was recently confirmed in a German study comparing irinotecan vs. best supportive care for gastric cancer [45] and in a South Korean study comparing salvage chemotherapy (docetaxel or irinotecan) plus best supportive care vs. best supportive care alone [46]. As the adverse reactions of trastuzumab alone were less toxic than those of the cytotoxic chemotherapies in the ToGA trial, the patient’s physical condition could be improved by minimizing adverse reactions caused by cytotoxic agents while using trastuzumab monotherapy to control disease progression. This improvement might also be attributed to the ease of performing second-line chemotherapy or further treatment, especially if there is a chemotherapy-free period before introducing second-line chemotherapy.

A case report highlighted the long-term survival (3 years and 6 months) of a Japanese patient included in the ToGA trial who had HER2-positive, unresectable advanced gastric cancer. This patient received trastuzumab plus capecitabine and cisplatin as initial therapy and continued maintenance therapy with trastuzumab alone for 47 cycles after completing 6 cycles of chemotherapy [47].

The German HerMES non-interventional observational study evaluated the efficacy, safety, and feasibility of trastuzumab in untreated patients with HER2-positive metastatic gastric cancer. In an interim analysis of 165 patients treated with regimens containing trastuzumab between April 2010 and April 2012, 20 % of patients continued trastuzumab alone after six cycles of chemotherapy, while >40 % of these patients received trastuzumab alone after ten cycles of chemotherapy, prompting the author to speculate that maintenance therapy with trastuzumab after chemotherapy is a preferred treatment option [48]. The median progression-free survival in that study was 6.9 months, and the overall rate of adverse events was 30.3 %. Grade 3–5 adverse events were rare, and the most common included vomiting (1.82 %) and general physical health deterioration (1.82 %).

Several other studies in breast cancer have also shown favorable responses to trastuzumab monotherapy [49, 50], which suggests that similar outcomes may be evident in gastric cancer.

Regarding the rationale for continuation of trastuzumab monotherapy, the ToGA (as shown in Fig. 3) and HERA trials demonstrated that continuous treatment with a single agent was capable of inhibiting HER2-positive tumor growth. However, this interpretation needs support from prospective comparative studies. To reproduce the effect of trastuzumab on the overall survival benefit observed in the ToGA trial, it is recommended that trastuzumab monotherapy be continued after completing chemotherapy in routine clinical practice.

## Should trastuzumab be continued beyond progressive disease?

Another important clinical issue is whether or not to continue molecular-targeted drugs upon disease progression. Currently, anticancer drugs, particularly cytotoxic drugs, are generally discontinued upon disease progression, and the patients started on subsequent aggressive treatment. Is there any evidence to support the validity of this approach, or should the molecular-targeted drug be continued?

Currently, no studies have examined this approach in the context of gastric cancer. However, several studies have been conducted that demonstrated the efficacy of continuing molecular-targeted drugs beyond disease progression in patients with breast cancer and colorectal cancer [51–56]. For example, it was recently reported that continuation of bevacizumab plus fluoropyrimidine-based chemotherapy beyond first progression in patients with metastatic colorectal cancer prolongs overall survival and progression-free survival compared with chemotherapy alone in the second-line setting [51].

Perhaps the best support for this concept comes from a randomized study performed in Germany in HER2-positive breast cancer [56]. In this study, patients with disease progression during trastuzumab-based therapy were randomly assigned to either capecitabine plus trastuzumab or capecitabine alone. The median time to progression (8.2 vs. 5.6 months; *P* = 0.0338) and overall survival (25.5 vs. 20.4 months; *P* = 0.257) were longer, and overall response (48.1 % vs. 27.0 %; *P* = 0.0115) was greater in patients who continued trastuzumab beyond disease progression. These results provide an answer to the clinical question “Is it effective to continue anti-HER2 therapy on disease progression in patients with metastasis or recurrence of HER2-positive breast cancer while undergoing trastuzumab therapy or after completing the therapy?” posed by the Japanese Breast Cancer Society [57].

The results of these studies suggested that continuing trastuzumab after progressive disease is a viable option, although larger, prospective studies are needed to confirm this approach. Now, several clinical trials in Japan (e.g., [58, 59]) are underway or are being planned to test this approach.

## Is there potential for using trastuzumab as perioperative chemotherapy in gastric cancer?

Perioperative chemotherapy is increasingly being considered as part of the treatment of various cancers, as it should allow earlier delivery of systemic treatment to the target lesion. Numerous studies have already shown favorable outcomes of perioperative chemotherapy compared with surgery alone or postoperative chemotherapy in the context of colorectal liver metastasis [60]. In terms of gastric cancer, Cunningham et al. [61] reported that a perioperative regimen consisting of epirubicin, cisplatin, and infused fluorouracil decreased tumor size and stage and improved progression-free and overall survival compared with surgery alone in patients with gastric cancer. Several case reports have documented favorable outcomes of trastuzumab as part of perioperative chemotherapy for gastric cancer [62, 63]. Both of these patients had complete pathological response after trastuzumab-based chemotherapy. Postmarketing clinical trials are now underway in Spain and Germany to examine the efficacy of perioperative adjuvant chemotherapy with trastuzumab in patients with HER2-positive gastric cancer (e.g., [64, 65]). The results of these studies should support an indication for trastuzumab as part of a perioperative chemotherapeutic regimen for treating HER2-positive gastric cancer.

## Future prospects: clinical development of new anti-HER2 drugs

Based on our increasing knowledge of the role for HER2 in gastric cancer, other agents targeting HER2 are also being developed for use in this setting, including lapatinib [66, 67]. Lapatinib is an orally active synthetic drug [68, 69] that is approved in Japan for HER2-positive breast cancer in combination with capecitabine [70]. Lapatinib inhibits HER2 signaling by blocking tyrosine kinase activity. In the Lapatinib (Tykerb) with Paclitaxel (Taxol) in Asian ErbB2+ (HER2+) Gastric Cancer Study (TYTAN), for example, patients across five Asian countries are to be randomly assigned to lapatinib (1,500 mg daily) plus paclitaxel (80 mg/m^2^ weekly) or paclitaxel alone. The primary endpoint of the study is overall survival. This study did not show an improvement in the primary endpoint. However, the efficacy of lapatinib was strongly suggested in the IHC+3 subset. These results indicate that the definition of HER2-positive gastric cancer is very important for the development of new anti-HER2 drugs [66]. Promising results have also been obtained for other compounds, including trastuzumab emtansine (T-DM1) [71] and pertuzumab [72], in HER2-positive breast cancer.

T-DM1 is an antibody-drug conjugate in which trastuzumab is conjugated to a cytotoxic compound, emtansine (DM1) [73]. T-DM1 combines the mode of action of trastuzumab with the targeted delivery of a potent cytotoxic. Upon binding of the trastuzumab moiety to HER2, T-DM1 is internalized into the tumor cell, releasing the DM1 moiety, which inhibits microtubules. A trial is now underway to examine the efficacy and safety of T-DM1 compared with standard taxane therapy in patients with HER2-positive gastric cancer [74]. In this study, patients will be randomized to one of three groups, 3.6 mg/kg T-DM1 every 3 weeks, 2.4 mg/kg T-DM1 every week, or standard taxane therapy, for at least four cycles (12 weeks). Planned endpoints include overall survival, progression-free survival, duration of response, and time to gastric cancer symptom progression, as well as safety.

Pertuzumab is a monoclonal antibody that prevents dimerization of HER2 with other HER receptors [75]. Its efficacy in combination with trastuzumab in patients with HER2-positive metastatic breast cancer has been demonstrated in a phase III clinical trial [72].

The results of these studies are eagerly awaited to examine the efficacy of this approach in patients with gastric cancer.

## Conclusions

In conclusion, this review has discussed just some of the abundant data showing the clinical benefits of trastuzumab for treating HER2-positive breast cancer, including prolonging overall survival and progression-free survival, and achieving greater clinical responses. The realization that HER2 is also overexpressed and/or gene-amplified in other forms of cancer, notably gastric cancer, has prompted studies in this setting, and the results accumulated to date indicate that trastuzumab is effective and tolerable in this setting. Based on the results of the ToGA trial, an extended indication for trastuzumab in gastric cancer was approved in Japan in March 2011. The clinical evidence suggests that trastuzumab monotherapy could be continued, even in patients who need to discontinue chemotherapy because of adverse reactions. More data are still needed, and studies are currently underway to confirm whether trastuzumab should be continued after disease progression. The introduction of trastuzumab led to the establishment of a new disease entity, “HER2-positive gastric cancer,” similar to HER2-positive breast cancer. It is expected that more anti-HER2 drugs will be developed and introduced into clinical practice to treat patients with HER2-positive cancers, including gastric cancer.
